# Reconstruction of Self-Sparse 2D NMR Spectra from Undersampled Data in the Indirect Dimension^†^

**DOI:** 10.3390/s110908888

**Published:** 2011-09-15

**Authors:** Xiaobo Qu, Di Guo, Xue Cao, Shuhui Cai, Zhong Chen

**Affiliations:** 1 Department of Communication Engineering, Fujian Key Laboratory of Plasma and Magnetic Resonance, Xiamen University, Xiamen 361005, China; E-Mails: quxiaobo@xmu.edu.cn (X.Q.); guodi@xmu.edu.cn (D.G.); 2 Department of Electronic Science, Fujian Key Laboratory of Plasma and Magnetic Resonance, Xiamen 361005, China; E-Mail: shcai@xmu.edu.cn (S.C.); 3 School of Software, Shanghai Jiao Tong University, Shanghai 200240, China; E-Mail: caoxue@sjtu.edu.cn (X.C.)

**Keywords:** NMR, spectral reconstruction, sparsity, undersampling, compressed sensing

## Abstract

Reducing the acquisition time for two-dimensional nuclear magnetic resonance (2D NMR) spectra is important. One way to achieve this goal is reducing the acquired data. In this paper, within the framework of compressed sensing, we proposed to undersample the data in the indirect dimension for a type of self-sparse 2D NMR spectra, that is, only a few meaningful spectral peaks occupy partial locations, while the rest of locations have very small or even no peaks. The spectrum is reconstructed by enforcing its sparsity in an identity matrix domain with *ℓ_p_* (*p* = 0.5) norm optimization algorithm. Both theoretical analysis and simulation results show that the proposed method can reduce the reconstruction errors compared with the wavelet-based *ℓ*_1_ norm optimization.

## 1. Introduction

Nuclear magnetic resonance (NMR) spectroscopy is widely utilized to analyze the structures of chemicals and proteins. Multidimensional NMR spectra can provide more information than one-dimensional (1D) NMR spectra. The acquisition time for a conventional two-dimensional (2D) NMR spectrum is mostly determined by the number of *t*_1_ increments in the indirect dimension. One possible way is to reduce the acquisition time is to reduce the number of *t*_1_ increments. However, this will result in aliasing of the spectrum in the indirect dimension [1,2], because the sampling rate is lower than the requirement of the Nyquist sampling rule.

Researchers have been seeking ways to suppress the aliasing from the aspects of sampling and reconstruction. Radial sampling presents relatively small leakage artifacts [3] and Poisson disk sampling is observed to provide a large low-artifact area in the signal vicinity [4]. The maximum sampling time for multi-dimensional NMR experiments was analyzed by Vosegaard and co-workers [5]. Besides the sampling patterns, some reconstruction algorithms have been employed to improve spectral quality, including maximum entropy [6,7], iterative CLEAN algorithm [8] and Bayesian reconstruction [9]. The sparse sampling was incorporated with intermolecular multiple-quantum coherences for high-resolution 2D NMR spectra in inhomogeneous fields [10].

Recently compressed sensing (CS) theory [11,12], for reconstructing signals from fewer numbers of measurements than the number that the Nyquist sampling rule requires has attracted lots of attention in medical imaging [13], single pixel imaging [14], and computer vision [15], *etc*. Under the assumption that the acquired data is sparse or compressible in a certain sparsifying transform domain, CS can successfully recover the original signal from a small number of linear projections with little or no loss of information. The choice of sparsifying transform is important in the CS. The sparsfying transform should be maximally incoherent with the measurement operator. Intuitively, the target signal should be sparsely represented in the transform domain, e.g., wavelet transform domain, and this spare representation should be spread out in the encoding scheme. Iddo introduced CS to reconstruct a 2D NMR spectrum from partial random measurements of its time domain signal under the assumption that the spectrum is sparse in the wavelet domain [16].

In this paper, we focus on the reconstruction of self-sparse NMR spectra, that is, a few meaningful spectral peaks occupy partial locations while the rest locations have very small or even no meaningful peaks. NMR spectra includes regions where no signals arise because of the discrete nature of chemical groups [17]. The reason we pay attention to self-sparse NMR spectra is that many NMR spectra of chemical substances fall in this type [3,10,16,17]. Based on the concept of sparsity and coherence in CS, we demonstrate that a wavelet transform is not necessary to sparsify the self-sparse NMR spectra or even worsens the reconstruction. We propose to reconstruct the NMR spectrum by enforcing its sparsity in an identity matrix domain with a *ℓ_p_* (*p* = 0.5) norm optimization algorithm. Simulation results show that the proposed method can reduce the reconstruction errors compared with the wavelet-based *ℓ*_1_ norm optimization.

Recently, Kazimierczuk and Orekhov [18] and Holland *et al.* [19] independently proposed to use CS in proton NMR and showed promising results in reducing acquired data. A combination of spatially encoding the indirect domain information and CS was proposed by Shrot and Frydman [20]. The spectra were considered to be sparse themselves [18–20], differing from the sparse representation using wavelets [16]. However, no comparison on the reconstructed spectra with and without wavelet transform was given and no theoretical analysis was presented. In this paper, we will analyze the performance of wavelet transform in the CS-NMR basing on the sparsity and coherence properties and simulated results.

The remainder of this paper is organized as follows. In Section 2, the reason to undersample the indirect dimension is given by calculating the acquisition time for a 2D NMR spectrum. In Section 3, the two key factors of CS, sparsity and coherence, are briefly summarized and their values are estimated for 2D spectra, followed by the proposed reconstruction method. In Section 4, reconstruction of self-sparse NMR spectra is simulated to show the shortcomings of the wavelet and the advantage of the identity matrix. The improvement of utilizing the *ℓ_p_* norm is also demonstrated. Finally, discussions and conclusions are given in Section 5.

## 2. Undersampling in the Indirect Dimension of 2D NMR

**Figure 1.**
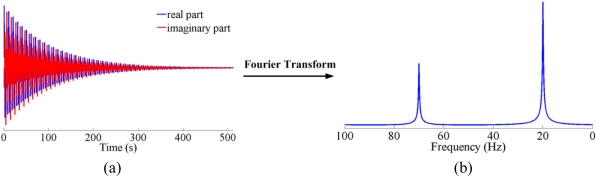
Simulated FID data in time domain (**a**) and its corresponding 1D NMR spectrum (**b**). Note: the FID is simulated according to Equation (1) with *J* = 2, *A*_1_ = 0.5, *A*_2_ = 1, Δ*t* = 0.01 s, *τ*_1_ = *τ*_2_ = 800, ∅_1_ = ∅_2_ = 0, and *ω*_1_ = 70 Hz, *ω*_2_ = 20 Hz.

In NMR spectroscopy, a typical sampled noiseless time domain signal can be described as a sum of exponentially decaying sinusoids:

(1) $$y_{k}=\sum_{j=1}^{J}\left(A_{j}e^{i\phi_{j}}\right)e^{-\frac{k\Delta t}{\tau_{j}}}e^{2\pi \mathrm{ik}\Delta t\omega_{j}}$$

where *J* is the number of sinusoids, *A_j_*, ∅*_j_*, *τ_j_* and *ω_j_* are the amplitude, phase in radians, decay time and frequency, respectively, of the *j*th sinusoid [21]. Δ*t* is the sampling interval and *k* (*k* = 0, 1, …, *K*) is an integer to denote the *k*th sample point. Such a signal will give rise to a spectrum that is the sum of Lorentizian peaks centered at different frequencies *ω_j_* [21], where *j* corresponds to *j*th type of nuclear spins. A conventional 1D single pulse NMR experiment enforces an excitation pulse on a sample followed immediately by data acquisition. The signal eventually decays due to relaxation [22], thus it is called *free induction decay* (FID). Fourier transform (FT) is applied on the FID to obtain a frequency domain spectrum. Figure 1 shows the simulated FID signal and the corresponding 1D NMR spectrum obtained from FT.

**Figure 2.**
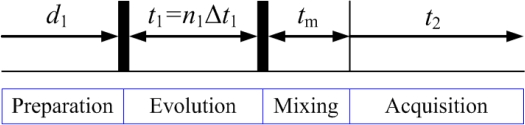
General scheme for 2D NMR spectra.

The typical experimental time for a 1D NMR spectrum usually takes several seconds, thus it is not time consuming. However, for a 2D NMR spectrum, the time domain signal is generated based on two time variables *t*_1_ and *t*_2_. As shown in Figure 2, one scan of 2D NMR spectrum contains three steps: first, the sample is excited by one or more pulses in the preparation period. These pulses result in the evolution of magnetization with time *t*_1_; then, the sample is further excited in the mixing period; finally, an FID signal is recorded as a function of *t*_2_. Usually, *t*_1_ is set as *t*_1_ = Δ*t*_1_, 2Δ*t*_1_, ..., *n*_1_Δ*t*_1_, *N*_1_Δ*t*_1_ (The increment Δ*t*_1_ is usually at the order of milliseconds). The number of *t*_1_ increments (*N*_1_) is determined by:

(2) $$N_{1}=\frac{{\mathrm{SW}}_{1}}{{\Delta f}_{1}}$$

where ${\mathrm{SW}}_{1}=\frac{1}{{\Delta t}_{1}}$ is the desired spectral width and ${\Delta f}_{1}=\frac{1}{N_{1}{\Delta t}_{1}}$ is the corresponding spectral resolution. The typical *N*_1_ is from 50 to 500 [22]. Given a fixed *t*_1_ = *n*_1_Δ*t*_1_, one scan is performed and the FID signal is recorded and stored along the direct dimension. After the scan, the nuclear spins are allowed to return to their equilibrium states before the next scan for *t*_1_ = (*n*_1_ + 1)Δ*t*_1_ [22].

Finally, 2D FT is performed on the 2D FID data. If the time for performing all the pulses in one scan is *t_p_*, the total scanning time for a 2D NMR spectrum will be:

(3) $$T_{N_{1}}=\sum_{n_{1}=1}^{N_{1}}\left(d_{1}+n_{1}{\Delta t}_{1}+t_{m}+t_{2}+t_{p}\right)=N_{1}\left(d_{1}+\frac{\left(1+N_{1}\right){\Delta t}_{1}}{2}+t_{m}+t_{2}+t_{p}\right)$$

In order to obtain a good resolution in the indirection dimension, *N*_1_ is usually several tens or hundreds or even more. This will cause the total scanning time for a 2D NMR spectrum to be tens of minutes or even several hours [22–26].

In this paper, we aim to reduce the scan number for the *t*_1_ dimension. Rather than using the uniform increment in the indirect dimension (*t*_1_ = Δ*t*_1_, 2Δ*t*_1_, ..., *n*_1_Δ*t*_1_, *N*_1_Δ*t*_1_), we randomly choose unduplicated *Q* numbers from *n_q_* ∈ {1, 2, ..., *N*_1_}, and let *t*_1_ = *n_q_*Δ*t*_1_. Let:

(4) $$\rho =\frac{Q}{N_{1}}$$

be the sampling rate in this paper, the total time to scan a 2D NMR spectrum is approximately:

(5) $$T_{Q}=\frac{Q}{N_{1}}T_{N_{1}}=\rho T_{N_{1}}$$

**Figure 3.**
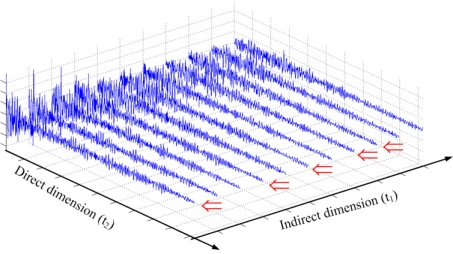
An example of random undersampling in the indirect dimension. The symbol ⇐ denotes the acquired FIDs.

The approximation is made by ignoring the total evolution time ∑_*n*_*q*_∈ {1,2,...,*N*_1_},*q* = 1,2,...,*Q*_*n_q_*Δ*t*_1_ since this value is only in the order of seconds. Compared to the time to acquire a 2D spectrum with fully sampled FIDs in the indirection dimension, undersampling the FIDs in the indirect dimension can greatly reduce the acquisition time for a 2D NMR spectrum if *ρ* is small enough. Figure 3 shows an example where we randomly undersample the indirect dimension with sampling rate *ρ* = 5/11 = 0.45. It means we save nearly half of the acquisition time of the conventional scheme.

However, this undersampling will result in aliasing artifacts [1,6]. It would be of great value if we can minimize these artifacts and reconstruct the full 2D NMR spectrum from the limited data. Here we explore the undersampling and reconstruction methods under the framework of CS.

## 3. Reconstruction of 2D Self-Sparse NMR Spectra with Compressed Sensing

### 3.1. Basic Concepts in Compressed Sensing

The CS proposed by Candès *et al.* [11] and Donoho [12] is a new theory to do undersampling and reconstruct the signal of interest from limited physically acquired data. They build a theoretical foundation that one can exactly or approximately recover signals from highly incomplete measurements. The two basic tenets to guarantee the performance of CS are sparsity and incoherence.

(a) Sparsity. For the signal **x** ∈ R*^N^* and a basis dictionary **Ψ** ∈ R^*S* × *N*^ (e.g., identity matrix, FT, discrete cosine transform or wavelet transform matrix), the sparsity is often interpreted as:

(6) $$S={\left\|\alpha \right\|}_{0}={\left\|\Psi x\right\|}_{0}\ll N$$

where ‖ **α** ‖_0_ denotes the *ℓ*_0_ norm that counts the nonzero entries in **α**, and *S* is the number of nonzero entries. If **x** is sparse without transformation (namely sparse in identity matrix **I** ∈ R^*N* × *N*^), it is called *self-sparse* since other complicated sparsifying transform, e.g., wavelet transform, is not required.

Candès *et al.* [11] and Donoho [12] proved that it is possible to recover the original signal **x** from O(*N*log*S*) measurements. This means the required number of measurements is proportional to the number of nonzero entries in the basis **Ψ**. The smaller the *S* is, the less the number of measurements is required.

(b) Incoherence. When a signal **x** is sampled by a sensing matrix Φ_*M* × *N*_, the measurements **y** ∈ R*^M^* of **x** is:

(7) f=Φx

The coherence is defined as [27,28]:

(8) $$\mu \left(\Phi ,\Psi \right)=\underset{k,j}{\text{max}}\left|\langle \phi_{k},\psi_{j}\rangle \right|$$

where ∅*_k_* is the *k*th rows of **Φ** and *Ψ_j_* is the *j*th column of **Ψ**. The coherence measures the largest correlation between any row of **Φ** and column of **Ψ**. The less the coherence between **Φ** and **Ψ** is, the smaller the *μ* is. The value range of *μ* is $\left[1,\sqrt{N}\right]$. The minimal coherence *μ =* 1 occurs when **Φ** and **Ψ** is a time-frequency pair [29]. CS requires the coherence to be as small as possible, which means each measurement vector ∅*_k_* must be ‘spread out’ in the **Ψ** domain [28].

If the signal **x** satisfies [30]:

(9) $$S={\left\|\alpha \right\|}_{0}<\frac{1}{2}\left(1+\frac{1}{\mu \left(\Phi ,\Psi \right)}\right)$$

it can be perfectly recovered by solving:

(10) $$\hat{\alpha }=\underset{\alpha }{\text{min}}{\left\|\alpha \right\|}_{0},\;\;\;s.t.\;\;\;y=\Phi \Psi \alpha$$

where ‖ **α** ‖_0_ denotes the *ℓ*_0_ norm that counts the nonzero entries in **α**.

The recovered signal is:

(11) $$\hat{x}=\Psi \hat{\alpha }$$

Equation (9) implies that if the coherence between **Φ** and **Ψ** is small, more non-zeros can be allowed in the sparse representation **α**. CS suggests **Φ** to be random enough to guarantee its incoherence with any **Ψ**. This is also observed that random sampling in time domain can improve the quality of reconstructed spectra [31].

However, *ℓ*_0_ norm is known to be intractable and sensitive to noise [11,12], and *ℓ*_1_ norm convex optimization is commonly used in CS to recover **x** by solving:

(12) $$\hat{\alpha }=\underset{\alpha }{\text{min}}{\left\|\alpha \right\|}_{1},\;\;\;s.t.\;\;\;y=\Phi \Psi \alpha$$

The accuracy of CS reconstruction using Equation (12) can be guaranteed if **ΦΨ** satisfies the appropriate restricted isometry properties [32]. A restricted isometry constant *σ*_s_ [32] defined as the smallest number such that:

(13) $$\left(1-\sigma_{S}\right){\left\|\alpha \right\|}_{2}^{2}\leq \left\|\Phi \Psi \alpha \right\|\leq \left(1+\sigma_{S}\right){\left\|\alpha \right\|}_{2}^{2}$$

holds for all vectors that have at most *S* nonzero entries. If $\sigma_{2S}<\sqrt{2}-1$, the solution to the *ℓ*_1_ norm problem is that of the *ℓ*_0_ problem [32].

The number of measurements *M* should satisfy:

(14) $$M\geq C\cdot \mu^{2}\left(\Phi ,\Psi \right)\cdot S\cdot \text{log}\;N$$

so that the signal **x** can be exactly recovered from measurements **y** in overwhelming majority of cases [28]. Equation (14) implies that the number of measurements is proportional to the number of nonzero entries *S* in **α** and the square of coherence *μ*. If both *S* and *μ* are small, the required number of measurements *M* could be small. This means that one can perform fewer measurements to save acquisition time while reconstruct original signal **x** very well.

Iddo [16] applied CS to remove the aliasing artifacts from incompletely acquired FID data by enforcing the sparsity of 2D NMR spectra in wavelet domain according to:

(15) $$\hat{\alpha }=\underset{\alpha }{\text{min}}{\left\|\alpha \right\|}_{1},\;\;s.t.\left\|y-\Theta F^{T}\Psi^{T}\alpha \right\|\leq \sigma$$

where **y** is the measurements in time domain, **Θ** is a random sampling operator defining the FIDs acquired in the indirect dimension, **F***^T^* denotes the inverse 2D FT, and **Ψ***^T^* is the inverse 2D wavelet transform. According to Equation (11), the recovered spectrum is x̂ = Ψ*^T^*α̂.

In this paper, we focus on the reconstruction of self-sparse NMR spectra in which significant peaks take up partial locations of the full NMR spectra while the remaining locations have very small or even no peaks. Ideally, if the number of sinusoids *J* in Equation (1) is very small, and the meaningful peaks are narrow enough relative to the whole 2D frequency coverage, the spectra can be considered to be sparse since the number of non-zeros for the spectra is much smaller than the number of spectrum points in the 2D NMR spectra.

The sparsifying transform and the coherence between **Ψ** and **Φ** = **ΘF***^T^* play important roles in the CS, as we have discussed. In the following sections, we will demonstrate that wavelet is not necessary to sparsify or even worsens the self-sparse NMR spectra based on the concept of sparsity and coherence. We will then reconstruct the NMR spectrum by enforcing its sparsity in an identity matrix domain with *ℓ_p_* (*p* = 0.5) norm optimization algorithm.

To represent the NMR spectra in conventional way [4–7,17], the X and Y coordinate axes are shown with unit of parts per million (ppm) [21] defined as:

(16) $$\delta =\frac{\omega -\omega_{\text{ref}}}{\omega_{0}}\times 10^{6}$$

where *δ* is the chemical shift of a peak with frequency *ω, ω*_ref_ is the frequency of a reference peak and *ω*_0_ is the spectrometer carrier frequency.

### 3.2. Sparsity of Self-Sparse NMR Spectra

**Figure 4.**
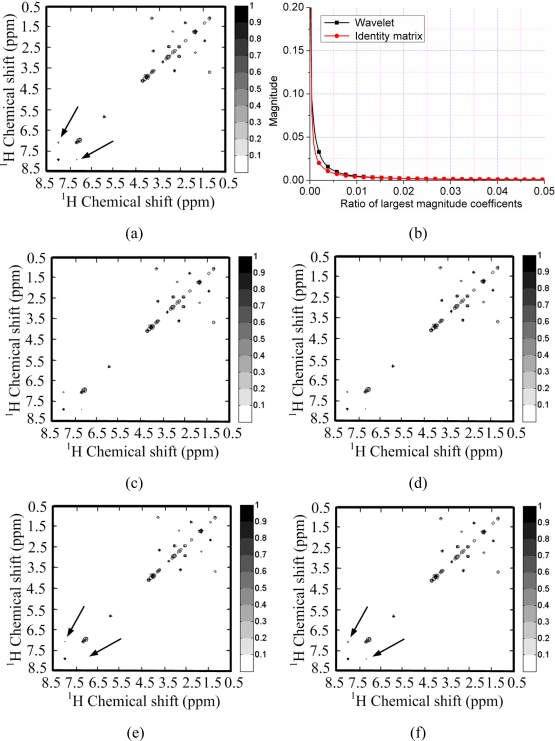
Sparsity of a ^1^H-^1^H COSY spectrum and its wavelet (symmlet wavelet with four decomposition levels and eight vanishing moments) representation. (**a**) The fully sampled NMR spectrum; (**b**) decay of real part of spectrum and its wavelet coefficients; (**c**,**e**) reconstructed spectra from 3% and 1% largest coefficients in wavelet domain; (**d**,**f**) reconstructed spectra from 3% and 1% largest coefficients in identity matrix domain. Note: the wavelet fails to represent peaks marked with arrows in (e) and these peaks are successfully represented in (f).

Figure 4(a) shows a 2D ^1^H-^1^H correlation spectroscopy (COSY) spectrum where most of the peaks fill partial and very limited regions of the full spectrum. This leads to the sparsity of spectrum because the number of non zeros in the 2D spectrum is much smaller than the number of spectrum points. This phenomenon is also observed by Yoh Matsuki *et al.* [17].

To test the sparsity of NMR spectra, we can measure the decay of coefficients in a sparsifying transform domain and evaluate the approximation error by retaining the *k***-term** largest coefficients, because the reconstruction error is proportional to the power law decay *k*^−*r*^, where *r* is a constant implying the sparsity of signal [29]. Rapid decay of coefficients implies that one can use less non-zero coefficients to approximate a NMR spectrum. If we directly measure the decay of signal without complicated sparsifying transform, e.g., wavelets, it means measure the self-sparsity of signal. Mathematical saying is measuring its sparsity in the identity matrix.

As shown in Figure 4(b), both the spectra and its wavelet coefficients can achieve rapid decay. By retaining 3% largest magnitude coefficients, the spectra can be reconstructed well in Figure 4(c,d). However, the spectrum is sparser than its representation in the wavelet domain. This is demonstrated by the faster decay of spectrum than that of its wavelet coefficients in Figure 4(b). By retaining the 1% largest magnitude coefficients, the wavelet fails to represent some peaks while the spectrum itself can represent these peaks, as marked by the arrows in Figure 4(e,f).

**Figure 5.**
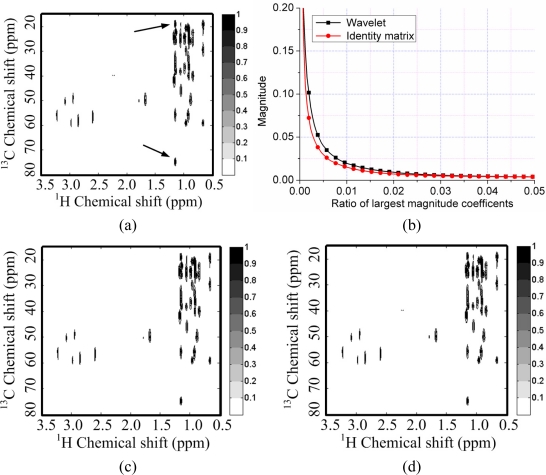

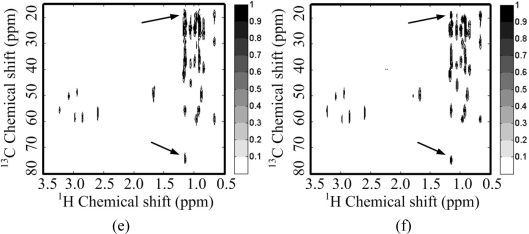
Sparsity of a ^1^H-^13^C COSY spectrum and its wavelet (symmlet wavelet with four decomposition levels and eight vanishing moments) representation. (**a**) The fully sampled NMR spectrum; (**b**) decay of real part of spectrum and its wavelet coefficients; (**c**,**e**) reconstructed spectra from 1% and 0.1% largest coefficients in wavelet domain; (**d**,**f**) reconstructed spectra from 1% and 0.1% largest coefficients in identity matrix domain. Note: the wavelet fails to represent peaks marked with arrows in (e) and these peaks are successfully represented in (f).

For a 2D ^1^H-^13^C COSY spectrum, the spectrum decays faster than its wavelet coefficients (Figure 5(b)). This implies that the identity matrix can provide a sparser representation of spectra than a wavelet does. Peaks are lost or distorted by using the wavelet transform to represent the spectrum (Figure 5(e)), but the spectrum is represented very well with the identity matrix (Figure 5(f)). This phenomenon is consistent with the observation on the 2D ^1^H-^1^H COSY spectrum discussed above.

As a result, this spectrum is self-sparse, which means spectrum is sparse in the identity matrix. Thus, according to Equations (9) and (14), it is better to use an identity matrix than to use a wavelet to reconstruct the self-sparse spectra from undersampled FIDs since the wavelet cannot provide a sparser representation of the spectrum. In fact, Stern *et al.* [33] proposed to do iterative soft thresholding on the spectrum directly, not on wavelet coefficients, to recover one dimensional NMR spectra from the truncated FIDs. Although the sparsity of NMR spectra is not explicitly expressed in that work [33], the recovered spectrum is obtained from minimizing *ℓ*_1_ norm of spectrum, which implies enforcing the sparsity of the spectrum. The problem of their method is that truncation violates the random sampling scheme in CS and results in strong Gibbs ringing which is hard to suppress [29]. What is more, truncating the 1D FID is not necessary to save the time to scan a spectrum since scanning a 1D NMR spectrum is fast and only takes on the order of seconds.

### 3.3. Coherence Property of Wavelet-Based and Identity Matrix-Based CS-NMR Spectra

Besides the sparsity of signal, another key factor for CS is the coherence between **Φ** and **Ψ** According to Equations (9) and (14), fewer measurements are required for signal sampling system **Φ** if it is less coherent with **Ψ** and the signal has same sparsity for different **Ψ**.

Pioneering work on CS has pointed out that the coherence of a time-frequency pair is *μ*(**Φ**, **I**) = *μ*(**ΘF***^T^*, **I**) = 1 [28]. Thus, we only need to compute the coherence between undersampled Fourier operator **Φ** and wavelet basis **Ψ***^T^*.

**Figure 6.**
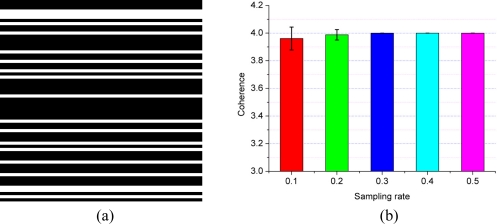
Coherence of wavelet and FT. (**a**) One sampling pattern in the indirect dimension with sampling rate *ρ* = 0.30 (fully sampled points in the indirect dimension is *N*_1_ = 64); (**b**) coherences for different sampling rates. The symmlet wavelet with four decomposition levels and eight vanishing moments is chosen as a typical wavelet for test, which is also the typical wavelet in [16]. Error bar stands for the standard deviation when repeating 10 times at each sampling rate.

The undersampling of **Θ** in the indirect dimension is carried out by choosing some of the FID points in this dimension. To make this undersampling intuitive, a binary mask which has the same size of 2D FID is shown as the undersampling pattern in Figure 6(a). If the value of mask at location (*i*, *j*) is equal to 1 shown as a white pixel, the FID at location (*i*, *j*) is acquired.

To avoid the influence of randomness on the coherence calculation, **Θ** is randomly generated 10 times and the coherence is averaged for each sampling rate. Figure 6(b) shows that the coherence between wavelet and undersampled Fourier operator **Φ** is larger than the coherence between identity matrix and **Φ**. So, from the aspect of coherence, it is also better to choose the identity matrix for self-sparse NMR spectra.

### 3.4. Reconstruction of Self-Sparse NMR Spectra with ℓ_p_ Norm Minimization

In this paper, we propose to reconstruct the self-sparse 2D NMR spectra with identity matrix **I** as follows:

(17) $$\hat{x}=\underset{x}{\text{min}}{\left\|x\right\|}_{1},s.t.\;\;\;y=\Phi x$$

where **Φ** = **ΘF***^T^*.

**Figure 7.**
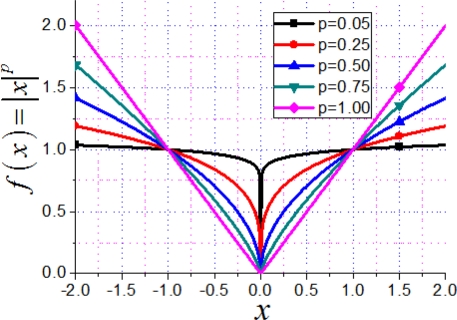
The value of *f*(*x*) = |*x*|*^p^* *versus* the value of *p*.

To further improve the reconstruction, a *ℓ_p_* (0 < *p* < 1) norm is incorporated which has been demonstrated to give better reconstruction of MR images with fewer measurements than *ℓ*_1_ norm does [34–37]:

(18) $$\hat{x}=\underset{x}{\text{min}}{\left\|x\right\|}_{p}^{p},s.t.\;\;\;y=\Phi x$$

where ${\left\|x\right\|}_{p}^{p}={\sum {}_{n=1}^{N}\left|x_{n}\right|}^{p}$ and *x_n_* is the *n***th** entry of vector **x**. For the function *f*(*x*) = |*x*|*^p^*, with *p* → 0, *f*(*x*) gets closer to the *ℓ*_0_ norm of *x*, as shown in Figure 7.

Theoretically, the required number of measurements [38] by enforcing the sparsity with a *ℓ_p_* (0 < *p* < 1) norm is:

(19) $$M\geq C_{1}\;\left(p\right)\;K+pC_{2}\;\left(p\right)\;K\;\text{log}\left(N/K\right)$$

where *C*_1_ and *C*_2_ are determined explicitly and bounded in *p* and the recommend *p* is 0.5 [34].

In this paper, the *ℓ_p_* norm minimization is solved via the *p*-shrinkage operator [39] with continuation algorithm [40] because of its fast computation. This algorithm is abbreviated as PSOCA and summarized in Algorithm 1.

**Algorithm 1.** Self-sparse NMR spectra reconstruction with undersampled data using PSOCA.

**Initialization:**
Input the sampled FID data **y**, set the regularization parameter *λ* =10^8^ and tolerance of inner loop *η* = 5 × 10^−3^. Initialize **x** = **FΘ***^T^***y**, **x_last_** = **x**, *β* = 2^6^, and **α** = **0**.
**Main:**
**While***β* ≤ 2^16^
**Inner loop:**
1. Given **x**,
**For***j* = 1 to *J*, solve Equation (20), the solution is **α**;
2. Given **α**, solve Equation (22), the solution is **x**;
3. If ‖Δ**x**‖ = ‖**x_last_** – **x**‖ > *η,***x_last_** ← **x**, go to step 1; Otherwise, go to step 4;
**Outer loop:**
4. **x̂** ← **x**, *β* ← 2*β*, go to step 1.
**End While**
**Output: x̂**

For a given continuation parameter *β*, PSOCA is implemented to solve two sub-problems:

(1) *p*-shrinkage operator

(20) $$\alpha_{j}=S_{ɛ}^{p}\left(x_{j}\right)=\text{max}\left\{x_{j}-ɛ{\left|x_{j}\right|}^{p-1},0\right\}\frac{x_{j}}{\left|x_{j}\right|}$$

where $ɛ=\beta^{\frac{1}{p-2}}$ and *β* is a parameter to be updated in the continuation scheme, **x***_j_* and **α***_j_* are the *j*th entry of column vectors **x** and **α**, respectively.

(2) solve the linear equation:

(21) $$\underset{x}{\text{min}}\frac{\beta }{2}{\left\|\alpha -x\right\|}_{2}^{2}+\frac{\lambda }{2}{\left\|y-\Phi x\right\|}_{2}^{2}$$

which can be simplified to:

(22) $$\left(\beta I+\lambda P\right)F^{T}x=\beta F^{T}\alpha +\lambda \Theta^{T}y$$

where the term **P** = **Θ***^T^***Θ** is a diagonal matrix consisting of ones and zeros. The diagonal entries of **P** correspond to the location of FID data and the entry value is 1 if a corresponding FID data point is sampled, otherwise the entry value is 0. Equation (22) can be solved fast since only a discrete Fourier transform and entry-wise division are required.

## 4. Simulation Results and Analysis

In this section, we will show the advantages of the proposed method in two aspects: (1) identity matrix as the sparsifying transform is compared with wavelet transform; (2) *ℓ_p_* norm minimization is compared with *ℓ*_1_ norm minimization. The recommended value of *p* is 0.5 for stability from empirical experiments [34]. The notation *ℓ*_0.5_ is short for *ℓ_p_* with *p* = 0.5. The typical *ℓ*_1_ norm minimization algorithms compared in this paper include iterative soft thresholding (IST) algorithm [16,41–43], alternating and continuation algorithm (ACA) [40]. The ACA is just *p* = 1 in PSOCA.

Because regions of small spectrum values usually contain no peaks for practical analysis, we set magnitude smaller than a constant *T* to be zero according to:

(23) $$x_{T}\;\left(j\right)=\{\begin{array}{ll} x\;\left(j\right), & x\;\left(j\right)\geq T\\ 0, & x\;\left(j\right)<T \end{array}$$

where **x** denotes the absolute value of spectra and **x***_T_* denotes the absolute value of post processed NMR spectra. For evaluation, *T* is set to two values. First, *T* is set to zero, which means a spectrum with small absolute values, possibly noise, are not suppressed. Second, *T* is set to the lowest value of contour when plotting the 2D spectrum. This is reasonable because peaks with absolute values smaller than *T* are not seen in the contour plot.

Suppose **x̂** denotes the reconstructed spectrum from undersampled FID, relative *ℓ*_2_ norm error (RLNE) is defined to measure the reconstruction error as:

(24) $$\text{RLNE }=\;\frac{{\left\|{\hat{x}}_{T}-{\tilde{x}}_{T}\right\|}_{2}}{{\left\|{\hat{x}}_{T}\right\|}_{2}}$$

where **x̃** is the reconstructed spectrum from fully sampled FID and 0 ≤ **x̃**, **x̂***_T_* ≤ 1. RLNE evaluates the normalized error presented in the reconstructed spectrum from undersampled FID. The lower the RLNE is, the better the reconstructed spectrum is consistent to the fully sampled spectrum.

### 4.1. Reconstruction of the spectra

**Figure 8.**
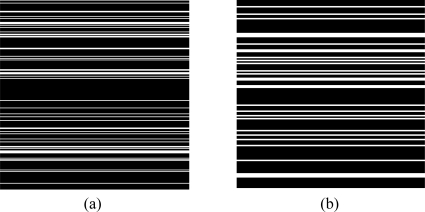
Sampling pattern used in simulation. (**a**) Cartesian sampling pattern with sampling rate 0.20 for the 2D ^1^H-^1^H COSY spectrum (*N*_1_ = 256 points) in Figure 4(a); and (**b**) Cartesian sampling pattern with sampling rate 0.25 for the 2D ^1^H-^13^C COSY spectrum (*N*_1_ = 128 points) in Figure 5(a).

The improvement by using the proposed method is verified from the less crowed ^1^H-^1^H COSY spectrum and more crowded ^1^H-^13^C COSY spectrum. The sampling patterns of the two spectra are shown in Figure 8.

**Figure 9.**
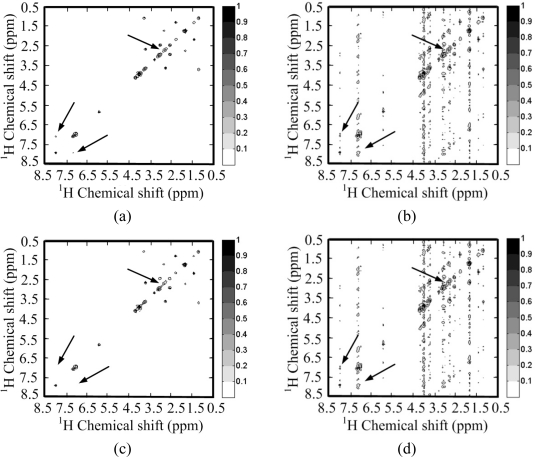

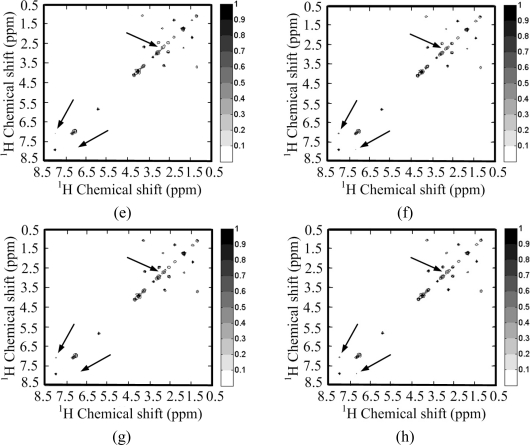
CS reconstruction of a 2D ^1^H-^1^H COSY spectrum using wavelet and identity matrix. (**a**,**b**) reconstructed spectra using fully sampled FID and undersampled FID with zero filling, respectively; (**c**,**d**) reconstructed spectra using wavelets and identity matrix with IST-based *ℓ*_1_ norm, respectively; (**e**,**f**) reconstructed spectra using wavelets and identity matrix with PSOCA-based *ℓ*_1_ norm, respectively; (**g**,**h**) reconstructed spectra using wavelets and identity matrix with PSOCA-based *ℓ_p_* norm, respectively.

Figure 9(c–h) show the reconstructed ^1^H-^1^H COSY spectra corresponding to the sampling pattern in Figure 9(a) with a sampling rate of 0.20. With the *ℓ*_1_ norm minimization, all the peaks are recovered successfully by using identity matrix (Figure 9(d,f)), while some peaks are lost by using wavelets (Figure 9(c,e)).

**Figure 10.**
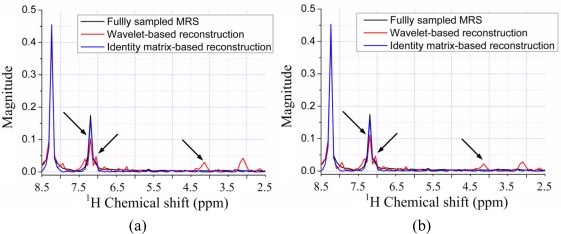

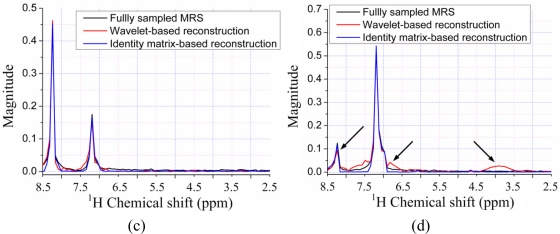
1D slices along the indirect dimension for the chemical shift of 8.2 ppm (**a**–**c**) or 7.2 ppm (**d**) in the direct dimension. (**a**) Spectra reconstructed with IST-based *ℓ*_1_ norm; (**b**) spectra reconstructed with PSOCA-based *ℓ*_1_ norm; (**c**) spectra reconstructed with PSOCA-based *ℓ*_0.5_ norm; (**d**) spectra reconstructed with PSOCA-based *ℓ*_0.5_ norm.

Since the contours for the marked peaks look faint, we also plot the 1D slices along the indirect dimension in Figure 10. The height of one peak in the wavelet-based reconstruction in Figure 10(a,b) are much lower than those in the fully sampled spectrum, leading to the peak lost in the contour plots in Figure 9(c,e).

Furthermore, the nonlinear operation on wavelet coefficients induces the artifacts labeled in Figure 9(c,e). This phenomenon is also observed in the 1D slices shown in Figure 10(a,b), where wavelet reconstruction generates illusive peaks. With the *ℓ*_0.5_ norm minimization, the errors caused from wavelet and identity matrix reconstruction are reduced, as shown in Table 1. One can still observe the reduced peak height and artifacts in wavelet-based reconstruction, but identity matrix performs very well (Figure 10(d)). The advantage of *ℓ*_0.5_ norm over *ℓ*_1_ norm is obvious in the crowded ^1^H-^13^C COSY spectra, as will be shown in the following discussion.

**Figure 11.**
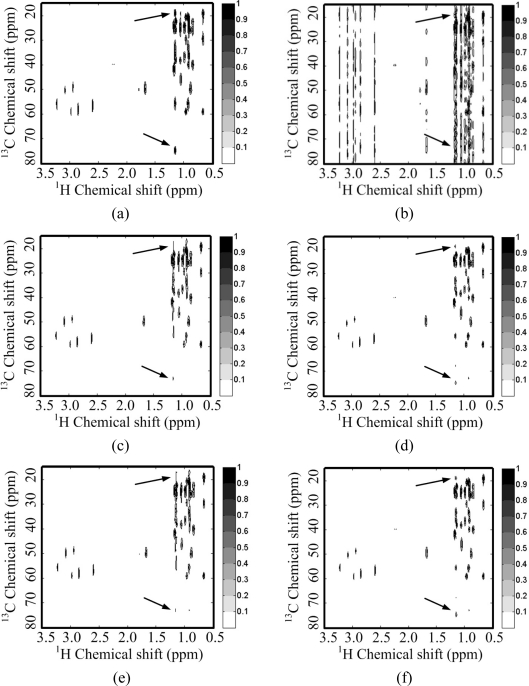

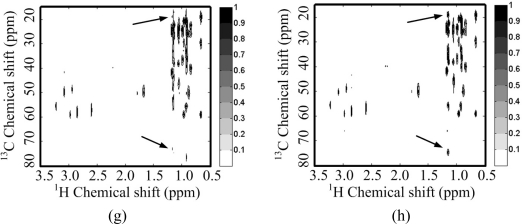
CS reconstruction of a 2D ^1^H-^13^C COSY spectrum using wavelet and identity matrix. (**a**,**b**) spectra reconstructed using fully sampled FID (*N*_1_ = 128 points) and undersampled FID with zero filling, respectively; (**c**,**d**) spectra reconstructed using wavelets and identity matrix with IST-based *ℓ*_1_ norm, respectively; (**e**,**f**) spectra reconstructed using wavelets and identity matrix with PSOCA-based *ℓ*_1_ norm, respectively; (**g**,**h**) spectra reconstructed using wavelets and identity matrix with PSOCA-based *ℓ*_0.5_ norm, respectively.

Figure 11 shows the reconstructed ^1^H-^13^C COSY spectra corresponding to the sampling pattern in Figure 8(b) with a sampling rate of 0.25. Some peaks are obviously lost in the reconstructed spectra using wavelets with both *ℓ*_1_ norm and *ℓ*_0.5_ norm minimization (Figure 11(c,e,g)). These lost peaks are found in the identity matrix-based reconstruction spectra (Figure 11(d,f,h)). With the *ℓ*_0.5_ norm minimization, the intensities of the peaks marked with arrow in Figure 11(h) are more consistent to the fully sampled spectra in Figure 11(b) than those in the reconstructed spectra with the *ℓ*_1_ norm minimization (Figure 11(d,f)). The smallest reconstruction error is achieved with the proposed identity matrix-based *ℓ*_0.5_ norm minimization method (Table 2).

All above simulation results demonstrate that wavelet-based reconstruction obviously induces the loss of some peaks in the crowded ^1^H-^13^C COSY spectrum and loss of some weak peaks in the less crowded ^1^H-^1^H COSY spectrum. The wavelet may even worsen the reconstructed spectra. Thus, it is not a good choice to use wavelets for the self-sparse spectra discussed in this paper.

### 4.2. Discussion on the Computation

Our simulation is run on a dual core 2.2 GHz CPU laptop with 3 GB RAM. The computational time for the algorithms using wavelet is two times that using the identity matrix, as shown in Table 3.

**Figure 12.**
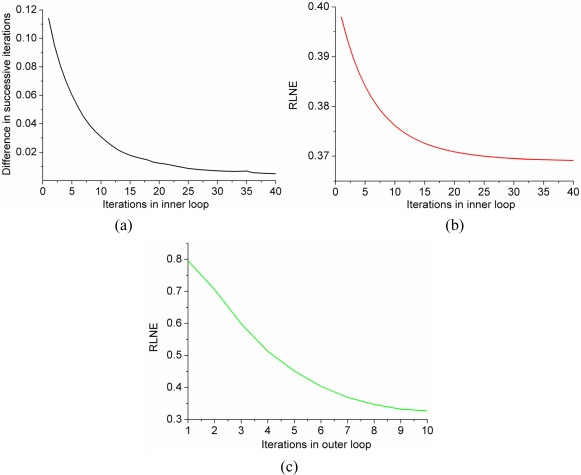
Numerical performance of PSOCA. (**a**) The *ℓ*_2_ norm of difference between reconstructed spectra in the current and previous iteration when *β* = 2^12^ in inner loop; (**b**) the reconstruction error RLNE of the reconstructed spectra when *β* = 2^12^ in inner loop; and (**c**) the reconstruction error RLNE *versus* the iterations in outer loop in PSOCA.

In the simulation, with the gradual increase of continuation parameter *β*, the previous solution was used as a ‘warm start’ for the next alternating optimization in the PSOCA. For a given *β*, with the increase of iterations in inner loop, the difference between reconstructed spectra decreases (see Figure 12(a)), so does the error between the reconstructed spectrum and the fully sampled spectrum (see Figure 12(b)). The reconstruction error decreases when *β* becomes large in the outer loop. The computational time of *ℓ*_0.5_ norm minimization in PSOCA is nearly four times as that of *ℓ*_1_ norm minimization, as shown in Table 3.

## 5. Conclusions and Future Work

Random sampling in the indirect dimension is introduced to reconstruct 2D self-sparse NMR spectra within the CS framework. Based on the assumption of sparsity of NMR spectra, one may remove the aliasing by penalizing the *ℓ*_1_ norm on the coefficients of the sparse representation of NMR spectra. Considering the sparsity and the coherence property, we demonstrate that wavelet transform may reduce the peak height and result in loss of peaks. Thus, a wavelet is not necessary and even worsens the reconstruction of self-sparse NMR spectra. With the *ℓ_p_* (*p =* 0.5) norm minimization, the quality of reconstructed spectra can be further improved.

However, how to define the meaningless peaks depends on applications and a qualitative analysis of self-sparse NMR spectra is needed in order to satisfy the requirement of CS. By defining regularity of ideal Lorentizian peaks with aspect to typical vanishing moment wavelet basis, it is possible to give a boundary for the approximation error of Lorentizian peaks in wavelet representation. Thus, one may quantify the sparsity of spectra composed of ideal Lorentizian peaks using wavelets. Another way is to set up a database and analyze the sparsity of the meaningful peaks based on the prior knowledge of chemists. Since the peak height may be reduced in the wavelet-based reconstruction and this reduction depends on the crowd of peaks, it is expected to give a quantitative analysis on the effect of using/skipping wavelet transform by setting up a simulated spectrum or spectrum from real chemical substance, in which the crowd of peaks and the fixed relative height of peaks are pre-defined in the spectrum. Besides, based on the coherence property in CS, the analysis of the performance of different random sampling schemes, e.g., Poisson disk sampling, may lead to further reduction of sampling rate and reconstruction error. Extension of the proposed method on higher dimensional NMR spectra is worth investigating.

## Figures and Tables

**Table 1.** Reconstruction error of a ^1^H-^1^H COSY spectrum.

**Methods**		**Zero-filling**	**IST *ℓ*_1_**	**PSOCA *ℓ*_1_**	**PSOCA *ℓ*_0.5_**
Wavelet	RLNE (T = 0)	2.054	0.415	0.393	0.430
	RLNE (T = 0.1)	0.059	0.012	0.010	0.007
Identity matrix	RLNE (T = 0)	2.054	0.282	0.273	0.245
	RLNE (T = 0.1)	0.059	0.010	0.007	0.022

**Table 2.** Reconstruction error of a ^1^H-^13^C COSY spectrum.

**Methods**		**Zero-filling**	**IST *ℓ*_1_**	**PSOCA *ℓ*_1_**	**PSOCA *ℓ*_0.5_**
Wavelet	RLNE (T = 0)	1.687	0.547	0.533	0.541
	RLNE (T = 0.1)	0.098	0.044	0.042	0.042
Identity matrix	RLNE (T = 0)	1.687	0.422	0.405	0.343
	RLNE (T = 0.1)	0.098	0.033	0.031	0.027

**Table 3.** Running time for reconstruction of a NMR spectrum (unit: second).

**Methods**	**Zero-filling**		**IST *ℓ*_1_**		**PSOCA *ℓ*_1_**		**PSOCA *ℓ*_0.5_**	

	^1^H-^1^H	^1^H-^13^C	^1^H-^1^H	^1^H-^13^C	^1^H-^1^H	^1^H-^13^C	^1^H-^1^H	^1^H-^13^C
Wavelet	0.1	0.1	11.1	56.8	8.5	70.4	29.1	221.2
Identity matrix	0.1	0.1	5.9	27.5	5.7	31.8	16.0	105.6
